# The effect of lack of ANC visit and unwanted pregnancy on home child-birth in Ethiopia: a systematic review and meta-analysis

**DOI:** 10.1038/s41598-022-05260-5

**Published:** 2022-01-27

**Authors:** Yitayish Damtie, Bereket Kefale, Melaku Yalew, Mastewal Arefaynie, Elsabeth Addisu, Tesfaye Birhan, Nigus Cherie, Bezawit Adane, Wolde Melese, Gedamnesh Bitew, Erkihun Tadesse, Reta Dewau, Atsedemariam Andualem

**Affiliations:** 1 Department of Reproductive and Family Health, School of Public Health, College of Medicine and Health Sciences, Wollo University, Dessie, Ethiopia; 2 Department of Epidemiology and Biostatistics, School of Public Health, College of Medicine and Health Sciences, Wollo University, Dessie, Ethiopia; 3 Department of Adult Health Nursing, School of Nursing and Midwifery, College of Medicine and Health Sciences, Wollo University, Dessie, Ethiopia

**Keywords:** Health care, Risk factors

## Abstract

Although extensive efforts were made to improve maternal and child health, the magnitude of home child-birth is considerably high in Ethiopia. Therefore, this meta-analysis aimed to estimate the effect of lack of ANC visit and unwanted pregnancy on home child-birth among reproductive-age women in Ethiopia. International databases, including Cochrane Library, Google Scholar, PubMed, Global Health, HINARI, and CINAHL were searched systematically to identify studies reporting the prevalence of home child-birth and its association with lack of ANC visit and unwanted pregnancy among reproductive-age women in Ethiopia. STATA/SE version-14 was used to analyze the data and Der Simonian and Liard's method of random effect model was used to estimate the pooled effects. The heterogeneity between study and publication bias was assessed by using I-squared statistics and Egger's test respectively. A total of 19 studies with 25,228 study participants were included in this meta-analysis. The pooled prevalence of home child-birth among reproductive-age women in Ethiopia was 55.3%. Sever heterogeneity was exhibited among the included studies (I^2^ = 99.8, p = 0.000). The odds of home child-birth among mothers who have no ANC visit was 3.64 times higher compared to their counterparts [OR = 3.64, 95%, CI: (1.45, 9.13)]. There was significant heterogeneity among the included studies (I^2^ = 94%, p = 0.000). However, there was no statistical evidence of publication bias in the pooled effect of lack of ANC visit on home child-birth (P = 0.302). Women who experienced unwanted pregnancy were 3.02 times higher to give birth at home compared to women with a wanted pregnancy [OR = 3.02, 95%CI: (1.19, 7.67)]. Severe heterogeneity was exhibited (I^2^ = 93.1%, p = 0.000) but, there was no evidence of significant publication bias in the pooled effect of unwanted pregnancy on home child-birth (P = 0.832). The proportion of home child-birth among reproductive-age women in Ethiopia remains high. Lack of ANC visit and unwanted pregnancy had a significant effect on the practice of home child-birth. Strengthening behavioral change communication programs should be the primary focus area to improve institutional delivery service utilization among women with lack of ANC visit and unwanted pregnancy.

## Introduction

Each year, an estimated 303,000 women die as a result of preventable pregnancy and childbirth complications worldwide^1^. Ninety-nine percent (99%) of the global maternal deaths occur in developing countries and Sub-Saharan African (SSA) countries alone shared 62% of all maternal deaths. Ethiopia is the fourth top ten countries in the world with the highest-burden of maternal mortality next to India, Nigeria, and the Democratic Republic Congo sharing 58% of all the global deaths^1^. The 2016 Ethiopia Demography and Health Survey (EDHS) report showed that the Maternal Mortality Ratio (MMR) in the country was 412/100,000 live births^2^.

More than two-thirds (73%) of maternal deaths occur during childbirth and within 24 h post-partum period due to direct obstetric causes such as hemorrhage, infection, obstructed labor, hypertension, and abortion^3^. Hypertensive disorders of pregnancy, hemorrhage, and sepsis alone accounted for more than 50% of all maternal mortality worldwide^4^.

One-third of births worldwide happen at home in the absence of SBA^5^. Studies have shown that home delivery ranged from 65% in Tanzania to 87.7% in Bangladesh^6^. The situation is worst in Africa in which more than 60% of women deliver at home without SBAs in contrary to less than 1% in developed nations^7^.

In Ethiopia, the prevalence of home delivery ranged from 19.1% in Shashemene town^8^ to 80% in Sherkole district of Benishangul Gumuz region^9^. According to the 2016 EDHS report, the prevalence of home delivery in the country was 73%, ranging from 3% in Addis Ababa to 85% in Afar region^2^.

In the last fifteen years, the Ethiopian government has established an inspiring framework for improving maternal and child health like training and deploying Health Officers with a master of Integrated Emergency Obstetrics and Surgery (IEOS) program, establishing Women Development Army (WDA), and creating pregnant women conferences to enable them to discuss issues related with pregnancy and delivery, constructing maternity waiting home at health facilities, availing traditional ambulances and increasing the number of modern ambulances for early referrals of emergency cases^10^. Regardless of these efforts, the rate of home delivery is considerably high in Ethiopia^2^.

Lack of ANC visits is the most fundamental and proximal factor responsible for the high rates of home delivery in developing countries like Ethiopia^11–14^. The World Health Organization (WHO) strongly emphasizes the need to have at least four ANC, in which the first visit should be started during the first trimester^15^. Although quality ANC strongly influences the use of skilled delivery care^16–18^, only 43% of women attended four or more ANC visits in Ethiopia^19^ which is far from the national target (increasing four or more ANC visits to 95% by the year 2020)^20^. Unwanted pregnancy is another major contributing factor affecting home childbirth^21,22^. A study showed that it increases the rate of home delivery by 40%^23^.

Reducing home delivery and increasing institutional delivery service utilization has a substantial contribution to the success of Sustainable Development Goal (SDG) 3 which aimed to reduce the global MMR below 70 per100, 000 live births by 2030^24^, and the Ethiopian Health Sector Transformation Plan (HSTP) IV which aimed to decrease the national MMR to 199 per 100,000 live births by 2020^20^.

Different studies were conducted to assess the practice of home child-birth in Ethiopia^8,9,15,25–40^. A systematic review and meta-analysis was also done^41^. However, the authors included those articles assessing institutional service utilization in their study which cannot indicate the exact figure of the prevalence of home delivery. They also included those studies assessing the intention of women towards the place of delivery that didn't clearly show the actual place of delivery. Moreover, they couldn't assess the effect of unwanted pregnancy on home delivery  although it is the major factor that affects the rate of home child-birth in the Ethiopian context. Therefore, this study aimed to estimate the effect of lack of ANC visit and unwanted pregnancy on home child-birth among reproductive-age women in Ethiopia. The result of this meta-analysis will help to identify whether the absence of ANC follow-up and unwanted pregnancy affect home delivery practice in Ethiopia and to reduce unwanted pregnancy by improving family planning utilization and promote the importance of ANC visit through information communication education and behavioral change communication programs accordingly.

## Materials and methods

### Searching strategy

The current systematic review and meta-analysis was organized according to the Preferred Reporting Items for Systematic Reviews and Meta-Analysis (PRISMA-2009) guideline^42^ (see supplementary file 1). International databases including Cochrane Library, Google Scholar, PubMed, Global Health, HINARI, and CINAHL were searched systematically to estimate the pooled prevalence of home child-birth and its association with lack of ANC visit and unwanted pregnancy among reproductive-age women in Ethiopia. The searching was carried out from September 1 up to December 30, 2020, by two authors (YD and BK) independently, and research articles published from 2000 up to December 30, 2020, were included in the analysis.

All the relevant studies were identified by using the following search terms: "proportion", "magnitude", "prevalence", "incidence", "home delivery", "home birth", "home child-birth", "risk factors" , "predictors", "factors", "determinants", "associated factors", "women", " mothers", " Reproductive Age Women", " women of child bearing age", "Ethiopia" using Boolean operators "OR" or "AND" (see supplementary file 2). The protocol of this meta-analysis was not registered on the International Prospective Register of Systematic Reviews (PROSPERO).

## Eligibility criteria

### Inclusion criteria

- **Population**: This systematic review and meta-analysis include studies conducted among women aged from15-49 years in Ethiopia.
- **Exposure**: ANC unbooked women and women with an unwanted pregnancies.
- **Comparison**: women who have ANC follow-up and women with a wanted pregnancy.
- **Outcome**: Studies assessed home child-birth as a primary outcome.
- **Study design**: all types of observational studies (Cross-sectional, case–control, and cohort) were included.
- **Country**: studies conducted only in Ethiopia.
- **Study setting**: all facility and community-based studies.
- **Time frame**: studies published from the beginning of 2000 up to December 30, 2020.
- **Publication**: both published and unpublished articles.
- **Language**: studies written in the English language were included in this study.

### Exclusion criteria

Those studies which are purely qualitative, the outcome of the interest not reported, and those with the absence of full text were excluded from this study after two email contacts of the corresponding author.

### Outcome measurement

This systematic review and meta-analysis measure three main outcomes. The primary outcome of this study was the pooled prevalence of home child-birth which was calculated from each primary article by dividing the number of women delivered at home to the total sample size multiplied by 100.

The second outcome was the association between lack of ANC visit and home child-birth. In this study, using ANC was considered if women received at least one ANC visit and more during the period of pregnancy. The third outcome of this study was the association between unwanted pregnancy and home child-birth. Unwanted pregnancy was defined as a pregnancy that occurred when no more children were desired. For the second and the third outcomes, the association between predictor variables (lack of ANC visit and unwanted pregnancy) and home child birth was determined in the form of the log odds ratio.

### Data extraction and quality assessment

After duplicate files were removed by endnote software, three reviewers (MY, NC, and MA) independently screened the leftover articles for inclusion. Then, based on their study design, the quality of the screened articles was assessed using Joana Brigg's Institute (JBI) critical appraisal checklist^43^. In this study, all of the included articles scored 50% and more, thus all are included in the review. A group of two authors (EA and BA) assessed the quality of each article independently and the disagreement or the difference in the results at the time of quality assessment was resolved by taking the mean score of the results of both reviewers.

All the relevant data were extracted by using a standardized data extraction sheet. The data extraction sheet includes the following variables; the name of the first author, region, year of study, publication year, study setting (whether it is institution or community based), study area, study design, the prevalence of home child-birth, sample size, response rate, the residence of the mother, number women delivered at home and frequencies of lack of ANC visit and unwanted pregnancy in the form of a two by two tables. Five reviewers (TB, ET, GB, WM, and AA) extract all the necessary data independently and the disagreement raised at the time of data extraction was resolved by consensus.

### Data analysis

All the necessary data were extracted using Microsoft Excel and exported to STATA version-14 for analysis. DerSimonian and Liard's method of random effect model (*p*-value < 0.05) was used to calculate the pooled prevalence of home child-birth in Ethiopia^44^. Besides, pooled odds ratios with 95% confidence intervals was used to determine the association of home child-birth with lack of ANC visit and unwanted pregnancy.

The variation among the original articles was statistically estimated by using the I^2^ test. I^2^ tests > 75% was considered as severe heterogeneity and was subjected to subgroup and univariate Meta-regression analyses to identify the possible source of variations among the point estimates of the individual studies. Moreover, the funnel plot and egger's regression test at a p-value less than 0.05 was used to assess the presence of publication bias^45^.

## Results

### Study selection

**Figure 1:**
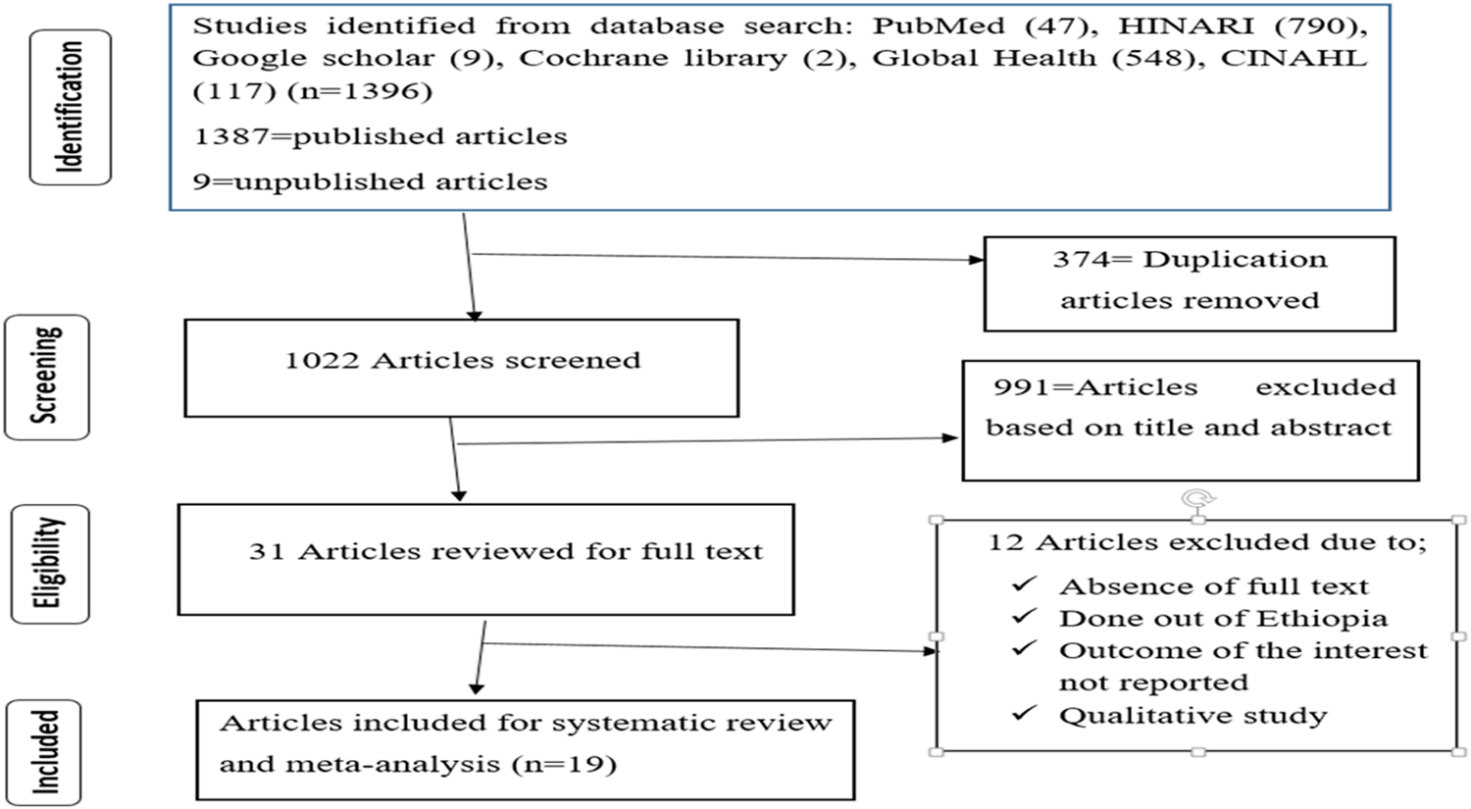
PRISMA flow diagram describing the selection of studies for systematic review and meta-analysis.

The review identified a total of 1,396 published and unpublished articles by searching international databases; Cochrane Library, Google Scholar, PubMed, Global Health, HINARI, and CINAHL. Then, 1,365 articles were removed due to duplication and as a result of their titles and abstracts. The rest 31 articles were critically assessed based on the eligibility criteria and finally, a total of 19 full-text articles were included in this meta-analysis (Fig. 1).

### Characteristics of the included studies

A total of nineteen studies^8,9,15,25–40^ with 25,228 study participants were included to estimate the pooled prevalence of home child-birth and its association with lack of ANC visit and unwanted pregnancy in Ethiopia. The sample size of the studies ranged from a minimum of 264 study participants from a study conducted in Abobo district, Gambella region^33^ to a maximum of 10,622 participants among a study conducted on 2016 EDHS data^32^. In this meta-analysis, seven regions from nine regions of the country were represented; six studies from SNNP^25,26,35,36,39,40^, four from Amhara^28,30,31,34^, three from Tigray^29,37,38^, one from Oromia^8^, one from Afar^27^, one from Benishangul Gumuz^9^ and one study from Gambella region^33^. However, there were no studies reported from Hareri, and Somali regions. Regarding the study design, twelve studies were cross-sectional^8,9,15,25–27,30–32,34,35,40^, five were case–control^28,29,33,36,37^ and the rest two were cohort studies^38,39^. (Table 1).

**Table 1:** Descriptive summary of nineteen studies included in the meta-analysis of home child-birth and associated factors among pregnant women in Ethiopia, 2021.

Authors	Publication year	Region	Study Area	Study design	Sample size	Response rate	No of women delivered at home	Prevalence (%)	Quality score (%)
Ibrahim S et al.^25^	2017	SNNPR	Anlemo	Cross-sectional	268	97.1	132	49.3	66.7
Gistane Ayele et al.^26^	2015	SNNPR	Arbaminch	Cross-sectional	436	90.6	346	79.4	77.8
Abdella M et al.^27^	2017	Afar	Ayssaita	Cross-sectional	317	99.7	225	71	77.8
Abebe et al.^28^	2012	Amhara	Bahirdar	Case–control	324	91	–	–	66.7
Berhe and Nigusie^9^	2020	Benishangul Gumuz	Sherkole	Cross-sectional	441	98	353	80	77.8
Tololu A et al.^29^	–	Tigray	Central zone	Case–control	300	95	–	–	66.7
Kasaye et al.^30^	2017	Amhara	Debremarkos	Cross-sectional	502	96.9	127	25.3	88.9
Wodaynew et al.^31^	2018	Amhara	Delanta	Cross-sectional	557	96.7	196	35.2	88.9
Chernet AG, et al.^32^	2019	EDHS based	EDHS 2016 based	Cross-sectional	10,622	NR	7137	67.2	88.9
Yebyo H et al.^15^	2015	EDHS based	EDHS 2011 based	Cross-sectional	7908	NR	6980	88.3	88.9
Mekonnen Y et al.^34^	2015	Amhara	Gozamin	Cross-sectional	497	100	374	75.3	88.9
DenekeDelibo et al.^35^	2020	SNNPR	Badawacho	Cross-sectional	531	96.2	391	73.6	88.9
Wondimu and Woldesemayat ^36^	2020	SNNPR	Hamar district	Case–control	292	94.5	–	–	66.7
Tsegay et al.^37^	2017	Tigray	Tanqua-Abergele	Case–control	275	96.5	–	–	66.7
Gultie et al.^8^	2016	Oromia	Shashemene	Cross-sectional	277	97.2	53	19.1	66.7
Hinsermu Bayu et al.^38^	2015	Tigray	Alamata, Mehoni, and Maichew	Cohort	465	89	134	28.8	77.8
Siyoum et al.^39^	2018	SNNPR	Wolaita	Cohort	505	91.2	68	13.5	77.8
Kucho and Mekonnen^40^	2017	SNNPR	Zala	Cross-sectional	447	100	302	67.6	77.8
Asmelash AberaMitiku et al.^33^	2020	Gambella	Abobo District	Case–control	264	100	–	–	66.7

SSNPR-Southern Nations, Nationalities, and Peoples Region.

### Prevalence of home child-birth in Ethiopia

**Figure 2:**
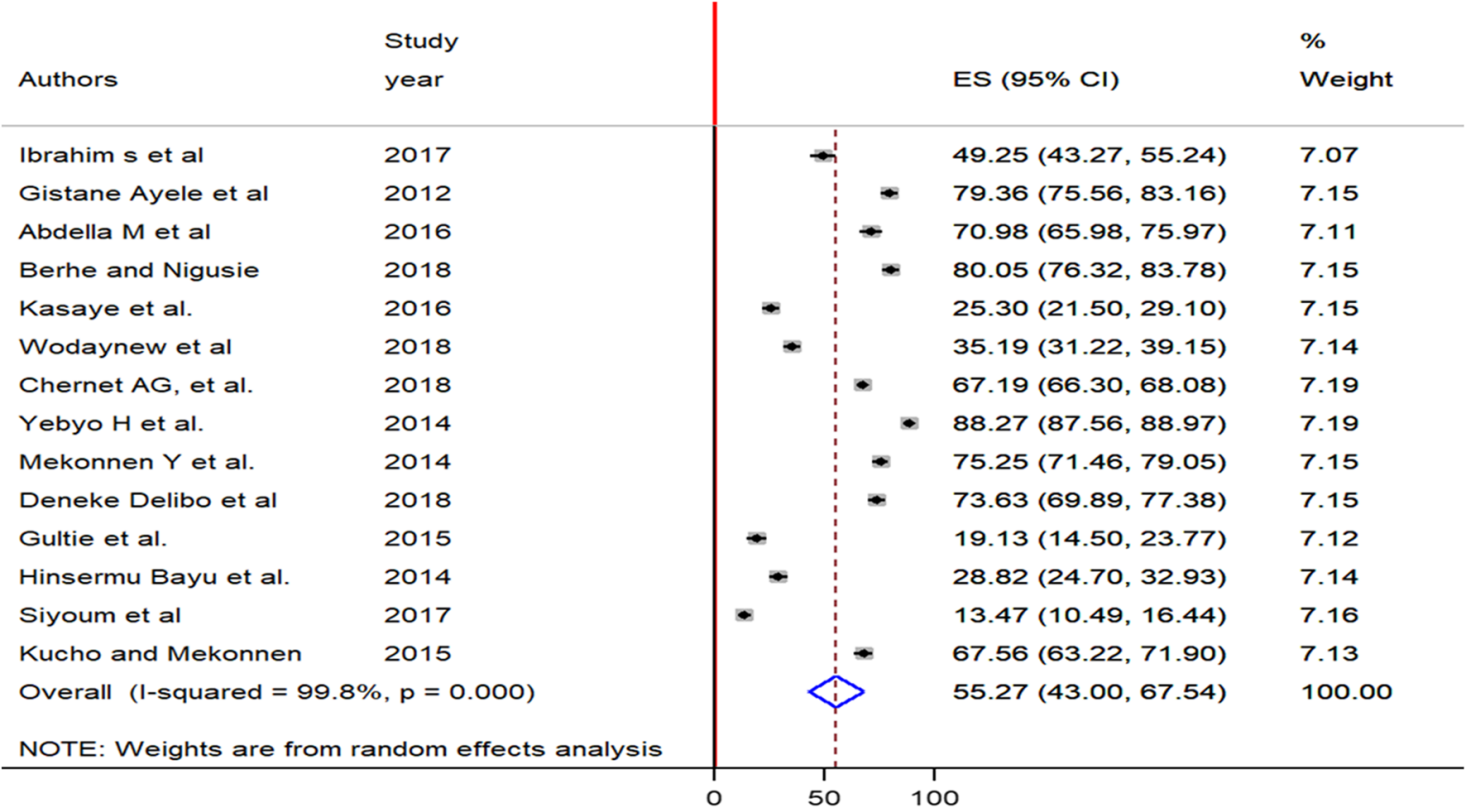
Forest plot of the pooled prevalence of home child-birth among reproductive-age women in Ethiopia, 2021.

A meta-analysis of 14 studies showed that the pooled prevalence of home child-birth among reproductive-age women in Ethiopia was 55.3% (95% CI: 43, 67.5). As it was shown on the figure, DerSimonian and Laird random-effects meta-analysis model was used to estimate the pooled effect as a result of severe heterogeneity between the included studies(I^2^ = 99.8, p = 0.000) (Fig. 2).

**Figure 3:**
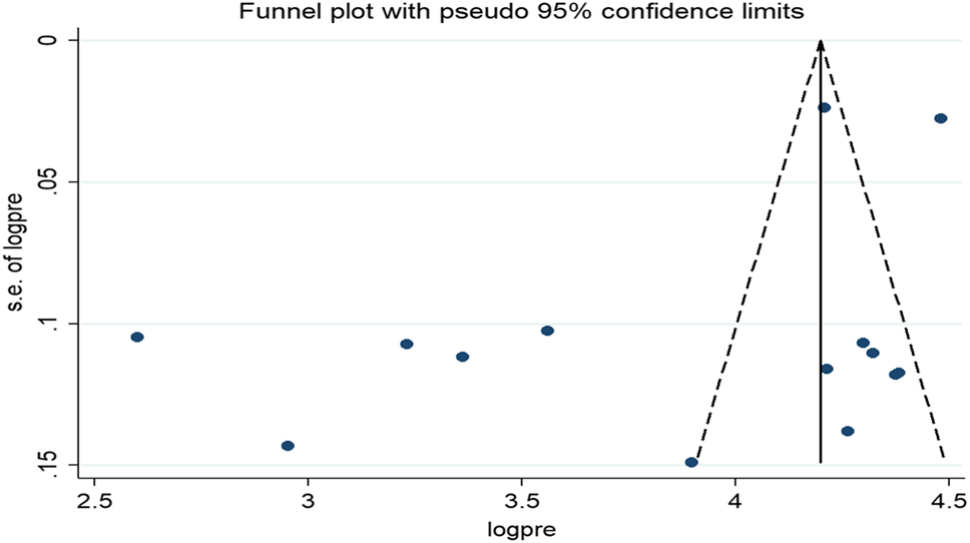
Funnel plot of the pooled prevalence of home child-birth among reproductive-age women in Ethiopia, 2021.

Publication bias was graphically assessed by using the funnel plot and there was an asymmetrical distribution of the effect estimates showing the presence of publication bias (Fig. 3), the eggers test statistics also evidenced that the presence of significant publication bias (*P* = 0.040). Duval and Tweedie’s 'trim and fill' method was used to reduce and adjust the effect of publication bias^46^, and there was no significant variation in the newly estimated pooled prevalence as compared to the previous one.

### Subgroup analysis and meta-regression

Subgroup analysis was conducted by region, residence, study design and sample size to identify the variation across the individual studies. Even though heterogeneity has still existed in the subgroup analysis of all the aforementioned parameters, the prevalence of home child-birth was significantly higher among studies conducted in rural settings [67.6%, 95% CI: (52.6, 82.6)] as compared to studies conducted in an urban setting. Similarly, the prevalence of home child-birth was significantly different among cross-sectional [60.9, 95% CI: (50.2, 71.8)] and cohort studies [21.1, 95%CI: (6.03, 36.1)] (Table 2).

**Table 2:** Subgroup prevalence of home child-birth among reproductive-age women in Ethiopia, 2021 (n = 14).

Variables	Characteristics	Included studies	Estimate (95% CI)	I^2^ (%)
Region	Amhara	3	45.2(14.9,75.5)	99.5
	SNNPR	5	56.6(28.4, 84.9)	99.6
	Others	6	59.2(44, 74.3)	99.8
Residence	Urban	2	24 (14.5, 33.5)	89.3
	Rural	3	67.6 (52.6, 82.6)	97.2
	Rural and urban	9	58.1( 43.4, 72.9)	99.8
Sample size	< 500	8	58.8(42.3, 75.4)	99.2
	≥ 500	6	50.5(31.6, 69.5)	99.9
Study design	Cross-sectional	12	60.9 (50.2, 71.8)	99.7
	Cohort	2	21.1( 6.03, 36.1)	97.1

SNNPR-Southern Nation Nationalities and Peoples Region; others- Benishangul Gumuz, Tigray, Afar, Oromia and EDHS based.

To identify the possible source of variation across the included studies, univariate meta-regression analysis was conducted using study-level characteristics (study year and sample size) as a cofactor, but none of them were found to be statistically significant (Table 3).

**Table 3:** Univariate meta-regression analysis to determine factors related to the heterogeneity of the prevalence of home child-birth in Ethiopia, 2021.

Variables	Coefficient	*P* value
Sample size	0.0028425	0.225
Study year	− 2.668666	0.492

### Factors associated with home child-birth

**Figure 4:**
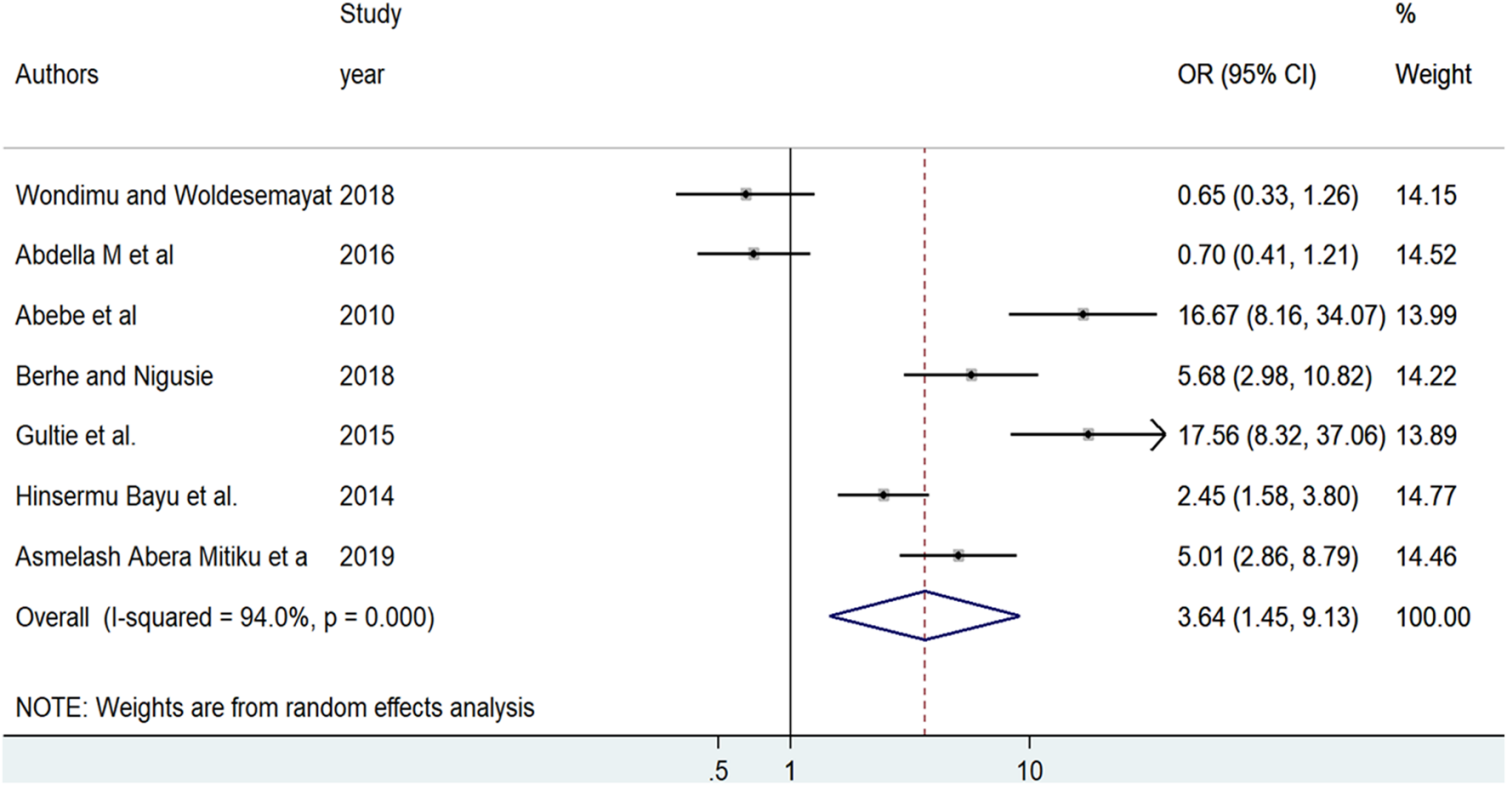
Forest plot of the pooled odds ratio of lack of ANC visit among reproductive age in Ethiopia, 2021.

The effect of lack of ANC visit on home delivery was examined based on the results of seven studies^8,9,27,28,33,36,38^. The analysis revealed that women who have no ANC visit were 3.64 times more likely to deliver at home as compared to women who have ANC visit [OR = 3.64, 95%, CI: (1.45, 9.13)]. A random-effect meta-analysis model was employed to examine the association between lack of ANC visit and home delivery as a result of significant heterogeneity among the included studies (I^2^ = 94%, p = 0.000) (Fig. 4).

**Figure 5:**
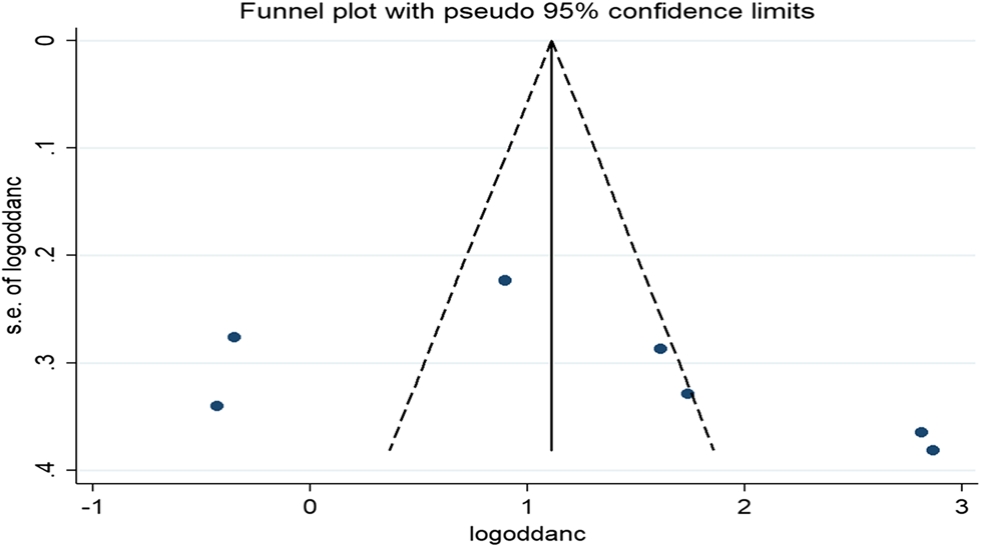
Funnel plot of the pooled odds ratio of lack of ANC visit among reproductive-age women in Ethiopia, 2021.

Publication bias was assessed using both funnel plots and Egger's tests. Although the funnel plot seems asymmetric (Fig. 5), the result of Egger's test witnessed that there was no statistical evidence of publication bias in the pooled effect of lack of ANC visit on home child-birth (P = 0.302).

**Figure 6:**
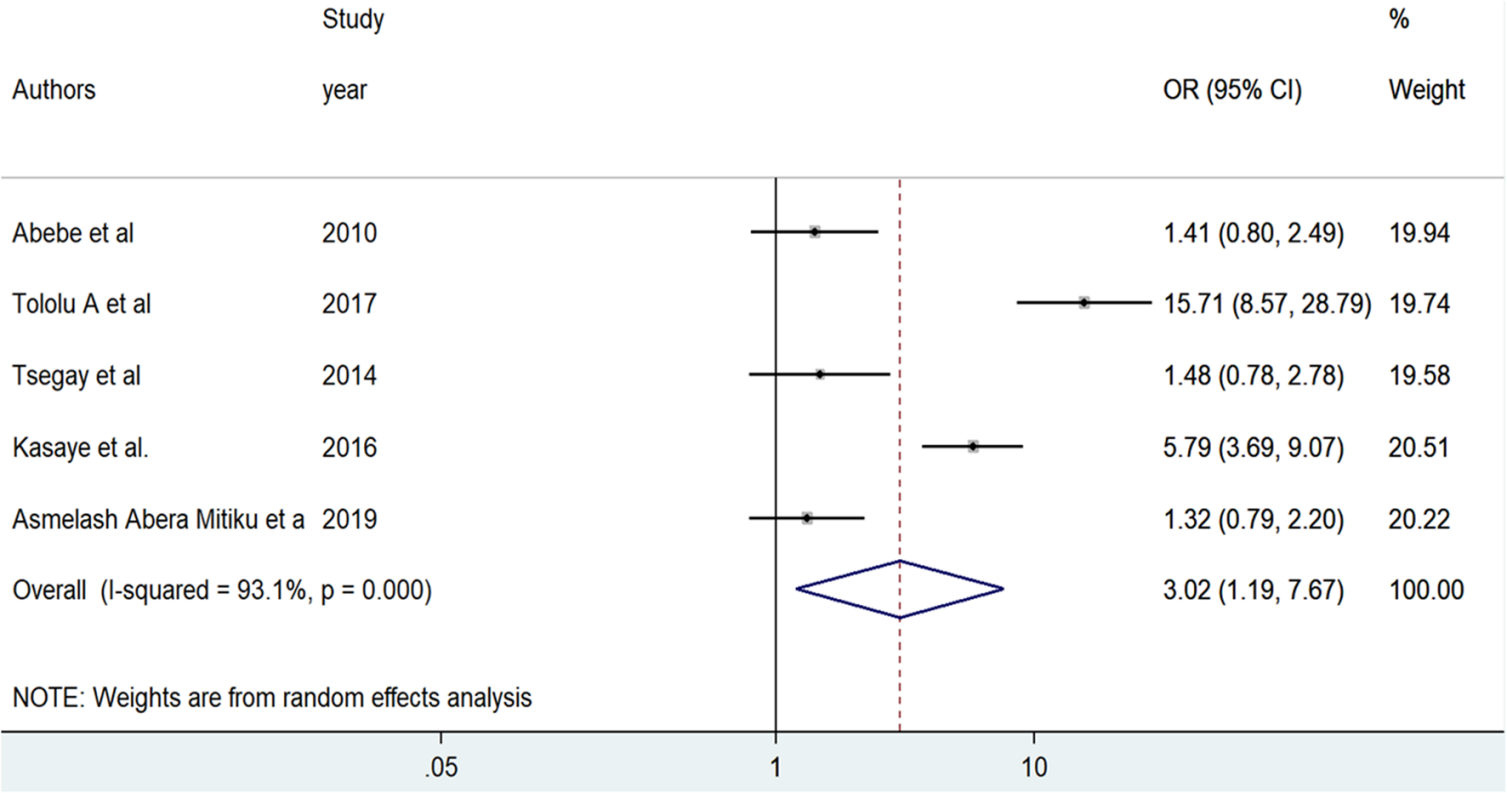
Forest plot of the pooled odds ratio of unwanted pregnancy among reproductive-age women in Ethiopia, 2021.

A total of five studies were included to identify the association between unwanted pregnancy and home delivery^28–30,33,37^. The random-effect meta-analysis revealed that women who experienced unwanted pregnancy were 3.02 times more likely to give birth at home as compared to women with wanted pregnancy [OR = 3.02, 95%CI: (1.19, 7.67)]. Sever heterogeneity was exhibited among the included articles (I^2^ = 93.1%, p = 0.000) (Fig. 6).

**Figure 7:**
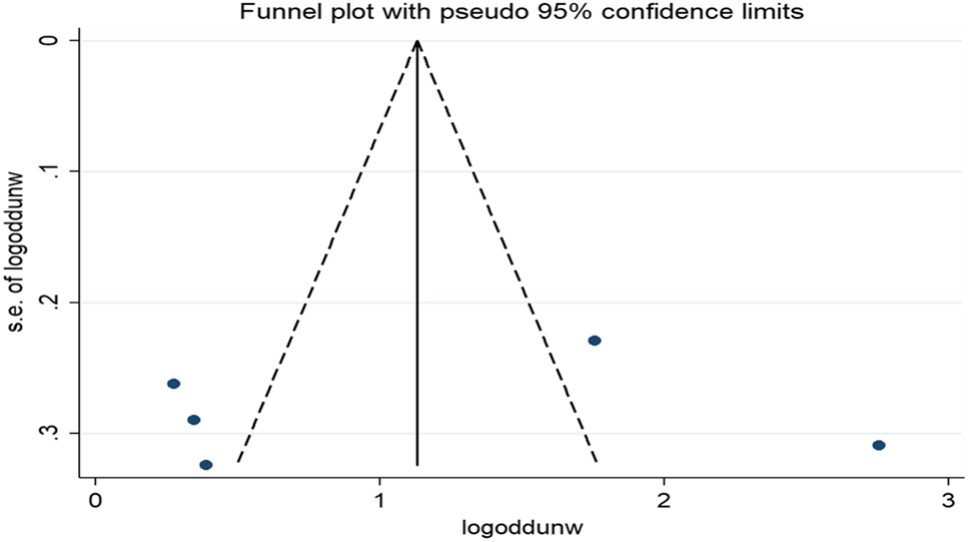
Funnel plot of the pooled odds ratio of unwanted pregnancy among reproductive-age women in Ethiopia, 2021.

There was no evidence of significant publication bias in the pooled effect of unwanted pregnancy on home delivery as it was indicated by Egger's tests (P = 0.832) despite the asymmetrical distribution of the effect estimates on the funnel plot (Fig. 7).

## Discussion

In this meta-analysis, the pooled prevalence of home child-birth among reproductive-age women in Ethiopia was 55.3% (95% CI: 43, 67.5). Lack of ANC visit and unwanted pregnancy had a statistically significant association with home delivery.

The pooled prevalence of home child-birth was in line with a recently published systematic review and meta-analysis study (66.7%)^41^. It is also in line with the 2019 mini Ethiopian Demographic and health survey report (50%)^19^ and another study conducted in Tanzania (65%)^47^. But, the finding was higher than studies conducted in Nepal(41.9%)^48^ and Burkina Faso (11%)^49^. The possible explanations for the variation could be due to the differences in the maternal socioeconomic and educational status, accesses to the health facility, quality of health service, media exposure, and health-seeking behavior. The result of subgroup analysis showed the pooled prevalence of home child-birth was significantly higher among studies conducted in rural settings as compared to studies conducted in an urban setting. The finding is similar to a systematic review and meta-analysis study conducted in sub-Saharan Africa^50^. It is because women living in rural areas face financial constraints, and lack transport access and decision-making power to receive skilled delivery care^51^. The finding of this meta-analysis indicates how much it is too far to achieve national and international goals to end maternal and infant mortality^20,24^. Thus, it urges the national government and other responsible individuals to invest their valuable effort to address the problem of home child-birth among women in all areas of the nation.

In this meta-analysis, the likelihood of home child-birth was higher among women who had no ANC visit compared to ANC-booked women. Consistent findings were also reported from studies conducted in Zambia, Malawi, and Kenya^52–54^. Studies evidenced that factors that predispose women not to use ANC also make them less likely to seek care during delivery^55,56^. ANC visit is the golden opportunity for pregnant women to discuss the place of delivery and to get information about the risks and complications encountered during pregnancy and delivery. A study conducted in Tanzania showed that women who are well informed about pregnancy complications during ANC visits are more likely to give birth in a health facility^57^. Failing to capitalize on ANC visits could be a significant missed opportunity to decrease maternal and neonatal mortality as a result of home delivery. Thus, clinicians need to promote the importance of ANC utilization to increase women's skilled delivery service utilization.

Unwanted pregnancy also had a positive association with home child-birth. It was in agreement with two studies conducted in Bangladesh^58,59^ and a study done in Zimbabwe^60^. A systematic review and meta-analysis conducted in low- and lower-middle-income countries showed that unwanted pregnancy was associated with a 25–39% reduction in the use of delivery care^61^. This could be because uncooperative and threatening behaviors from family members and sexual partners are common among women with an unwanted pregnancy and these might limit women’s decision-making power and financial support which might have a negative impact on accessing institutional delivery services^61,62^. In addition, mothers with unwanted pregnancies may not worried about the health of the baby and might give less value to the expected child so that they do not seek delivery care utilization.

As a limitation, this systematic review and meta-analysis may not be representative of articles published in languages other than English. In addition, most of the included articles were cross-sectional and had a small sample size and thus might affect the pooled effect. Moreover, this meta-analysis may not be representative of all regions of Ethiopia.

## Conclusions

The proportion of home child-birth in Ethiopia was considerably high. The lack of ANC visit and unwanted pregnancy had a significant effect on home delivery. Increasing the access to family planning services and strengthening the Information, Education and Communication (IEC) program is vital to decrease unwanted pregnancy and improve maternal health service utilization particularly ANC follow-up to make a home delivery-free community.

## Supplementary Information

Supplementary Information 1.

Supplementary Information 2.

Supplementary Information 3.

## Data Availability

The datasets used and/or analyzed during this study are available from the corresponding author on reasonable request.

## Abbreviations

ANC: Antenatal care
EDHS: Ethiopia demography and health survey
HSTP: Health sector transformation plan
IEC: Information, education and communication
IESO: Integrated emergency obstetrics and surgery
JBI: Joana Brigg's institute
MMR: Maternal mortality ratio
PRISMA: Preferred reporting items for systematic reviews and meta-analysis
SBA: Skilled birth attendants
SDG: Sustainable development goal
SSA: Sub-Saharan African
WDA: Women development army
