# Supplementary material for: The effect of lack of ANC visit and unwanted pregnancy on home child-birth in Ethiopia: a systematic review and meta-analysis
Source: Sci Rep. 2022 Jan 27;12:1490. doi: 10.1038/s41598-022-05260-5 (PMC8795397; doi:10.1038/s41598-022-05260-5)
Supplement: Supplementary file 2 — Supplementary Information 2. [file 41598_2022_5260_MOESM2_ESM.docx]

**Supplementary file 2:** Search strategy used to estimate the effect of lack of ANC visit and unwanted pregnancy on home child-birth in Ethiopia: a systematic review and meta-analysis

**PubMed (47)**

**(((((((proportion) OR (prevalence)) OR (magnitude)) OR (incidence)) AND ((("home birth") OR ("home childbirth"[MeSH Terms])) OR (home delivery[MeSH Terms]))) AND (((((factors) OR ("associated factors")) OR (determinants)) OR (predictors)) OR ("risk factors"))) AND ((((mothers) OR (women)) OR ("reproductive age women")) OR ("women of child bearing age"))) AND (Ethiopia)**

*Filter applied: Human, English, Female, and Adult: 19+ years, from 2000/1/1- 2020/12/30.*

***HINARI (790)***

***((proportion) OR (magnitude) OR (prevalence) OR (incidence)) AND (("home birth") OR ("home child birth") OR ("child birth at home") OR ("home delivery")) AND ((factor) OR ("associated factor") OR ("determinants") OR ("risk factor") OR (predictors)) AND ((women) OR (mother) OR ("reproductive age women") OR ("women of child bearing age")) AND (Ethiopia****) Filter applied: from 2000/1/1- 2020/12/30, female, human, English.*

**Google scholar** (9)

((proportion) OR (magnitude) OR (prevalence) OR (incidence)) AND (("home birth") OR ("home childbirth") OR ("childbirth at home") OR ("home delivery")) AND ((factor) OR ("associated factor") OR ("determinants") OR ("risk factor") OR (predictors)) AND ((women) OR (mother) OR ("reproductive age women") OR ("women of child bearing age")) AND (Ethiopia) *Filter applied: from 2000- 2020,* **Cochrane library (2)**

## proportion OR prevalence OR incidence OR magnitude in Title Abstract Keyword AND "home birth" OR "home childbirth" OR "childbirth at home" OR "home delivery" in Title Abstract Keyword AND factors OR determinants OR "associated factors" OR "risk factors" OR predictors in Title Abstract Keyword AND women OR mothers OR "reproductive age women" OR "women of child bearing age" in Title Abstract Keyword AND "Ethiopian" in Title Abstract Keyword - (Word variations have been searched)

## *Filter applied: from 2000- 2020.*

##

**Global health (548)**

(Proportion) OR (magnitude) OR ( prevalence ) OR (incidence) AND ("home childbirth" ) OR (" home delivery") OR ("home birth") and (risk factors) OR (determinants) OR ( "associated factors" ) (predictors) AND ("reproductive age women" ) OR ("women of child bearing age" ) OR ( women ) OR (mothers ) AND (Ethiopia ) AND yr:[2000 TO 2020]

**CINAHL (117)**

((proportion) OR (magnitude) OR (prevalence) OR (incidence)) AND (("home birth") OR ("home childbirth") OR ("childbirth at home") OR ("home delivery")) AND ((factor) OR ("associated factor") OR ("determinants") OR ("risk factor") OR (predictors)) AND ((women) OR (mother) OR ("reproductive age women") OR ("women of child bearing age")) AND (Ethiopia) *Filter applied: from 2000-2020*
